# *Helicobacter pylori* Infections in Children

**DOI:** 10.3390/antibiotics12091440

**Published:** 2023-09-12

**Authors:** Julie Nguyen, Kallirroi Kotilea, Patrick Bontems, Veronique Yvette Miendje Deyi

**Affiliations:** 1 Department of Pediatric Gastroenterology, Hopital Universitaire des Enfants Reine Fabiola, Université Libre de Bruxelles, 1000 Brussels, Belgium; 2 Belgian Helicobacter and Microbiota Study Group (BHMSG), 1000 Brussels, Belgium; 3 Department of Microbiology, Laboratoire Hospitalier Universitaire de Bruxelles-Brussel Universitair Laboratorium (LHUB-ULB), Université Libre de Bruxelles, 1000 Brussels, Belgium

**Keywords:** *Helicobacter pylori*, children, epidemiology, pathogenesis, clinical manifestations, diagnosis, antibiotic resistance and treatment

## Abstract

In the context of epidemiology, host response, disease presentation, diagnosis, and treatment management, the manifestation of *Helicobacter pylori* (*H. pylori*) infection diverges between children and adults. *H. pylori* infection stands out as one of the most prevalent bacterial infections globally, and its prevalence in both children and adults is decreasing in many developing countries but some still struggle with a high prevalence of pediatric *H. pylori* infection and its consequences. The majority of infected children are asymptomatic and pediatric studies do not support the involvement of *H. pylori* in functional disorders such as recurrent abdominal pain. The pathophysiology of *H. pylori* infection relies on complex bacterial virulence mechanisms and their interaction with the host immune system and environmental factors. This interaction gives rise to diverse gastritis phenotypes, which subsequently influence the potential development of various gastroduodenal pathologies. In clinical settings, the diagnosis of this infection in childhood requires an upper gastrointestinal endoscopic exam with mucosal biopsy samples for histology and culture, or Polymerase Chain Reaction (PCR) at the very least. When warranted, eradication treatment should be given when good compliance is expected, and there should be systematic use of a treatment adapted to the antimicrobial susceptibility profile. To combat the burgeoning threat of multidrug resistance, vigilant surveillance of resistance patterns and strategic antibiotic management are paramount.

## 1. Introduction

*Helicobacter pylori* (*H. pylori*) is a spiral-shaped and flagellated Gram-negative bacterium that specifically colonizes the human stomach. Its initial description can be traced back to 1983, credited to the work of Barry Marshall and Robin Warren [1].

In the last years, the annual count of registered publications has exceeded 1800, with over 100 of those focused on *H. pylori* in pediatrics.

This bacterium is generally acquired during childhood and continues throughout the life unless specific treatment is prescribed. It can lead to severe gastroduodenal pathologies, including chronic gastritis, peptic ulcer, gastric cancer, and gastric mucosa-associated lymphoid tissue (MALT) lymphoma.

The prevalence of *H. pylori* is largely associated with socioeconomic status and living conditions, particularly those marked by overcrowding and poor hygiene [2].

Children differ from adults and have a low rate of severe disease associated with *H. pylori* and almost an absence of gastric malignancies. Infection is generally asymptomatic in children. Several studies have shown a lack of evidence regarding the relation between abdominal pain or other abdominal symptoms in children and *H. pylori* infection [3]. Considering these distinctions, guidelines for pediatric care provided by numerous esteemed societies, including the European Society of Pediatric Gastroenterology Hepatology and Nutrition and the North American Society of Pediatric Gastroenterology, Hepatology, and Nutrition, advise against employing a ‘test and treat strategy’ for the management of *H. pylori* in children [4].

This review, drawing from recent and pertinent publications, seeks to provide a concise overview of *H. pylori* in pediatric populations, encompassing shifts in its prevalence, risk factors, pathogenesis, clinical manifestations, and discussing the appropriate utilization of relevant diagnostic methods and clinical strategies employed in patient care. Moreover, recent data on resistance rates of *H. pylori* to currently employed antibiotics are discussed, with a focus on primary resistance and its trend across diverse regions worldwide.

## 2. Epidemiology

The discovery of *H. pylori* DNA in Peruvian mummies and other well-preserved human corpses from prehistoric times suggests that the infection has existed in human communities for thousands of years [5]. *H. pylori* infection is spread worldwide, as there seems to be no population, even in the most remote corners of the world, whose members are exempt from *H. pylori*. Seven distinct populations and subpopulations of *H. pylori* are currently identified in the modern context. These modern populations trace their genetic origins back to ancestral groups that emerged in Africa, Central Asia, and East Asia. It is of great interest to note that modern worldwide distributions follow human migratory fluxes [6].

In 2017, a systematic review and meta-analysis with 184 reports from 62 countries showed that the prevalence of *H. pylori* infection in adults ranges from 24% to 73% across continents, with a pooled global prevalence estimated at more than 50%, depending on geographic location and economic development. According to regional estimates of prevalence, the global population of individuals infected with *H. pylori* was approximated at 4.4 billion in 2015. Africa exhibited the highest pooled prevalence of *H. pylori* infection (70.1%; 95% CI, 62.6–77.7%), followed by South America (69.4%; 95% CI, 63.9–74.9%) and Western Asia (66.6%; 95% CI, 56.1–77.0%). In contrast, Oceania (24.4%; 95% CI, 18.5–30.4%), Western Europe (34.3%; 95% CI, 31.3–37.2%), and Northern America (37.1%; 95% CI, 32.3–41.9%) have the lowest prevalence rates [7].

In children, a 2022 systematic review and meta-analysis encompassing 60 countries and involving 152,650 patients across 198 epidemiological studies in the past 30 years, yielded insights into the global prevalence of *H. pylori* infection. Employing a multilevel mixed-effects meta-regression methodology, this study established that the overall prevalence of *H. pylori* infection in children worldwide stands at 32.3% (95% CI, 27.3–37.8%), which varied by diagnostic test (28.6% [23.0–35.0] for serology vs. 35.9% [29.2–43.2] for urea breath tests or stool antigen tests) [8]. This prevalence displayed geographical disparities, which were noteworthy even within the confines of the same country. For example, the prevalence ranged from 3.8% to 9.5% depending on the prefecture in Japan. The prevalence of *H. pylori* is low (6.0%) in children from North–Central Nigeria, whereas it is relatively high (28%) in the Southwest part of the country [9]. Notably, the prevalence of *H. pylori* infection in children in low-income and middle-income countries (43.2%) is almost twice that in high-income countries (21.7%) [8].

Studies have consistently shown over the past three decades that there has been a significant decrease in the prevalence of *H. pylori* infection among children on a global scale, particularly in industrialized countries of the Western world. In contrast, prevalence has plateaued at a high level in developing and newly industrialized countries [10]. This declining prevalence is explained by the steady improvements in sanitation and living conditions from the 1950s. In addition, it was speculated that the decreasing birth rate might have led to fewer children per family, thus decreasing the risk of intra-familial infection [11].

In Portugal, we note a significant decreasing trend in the prevalence of *H. pylori* infection in children [12]. In China, the infection rate has decreased over the past decades from 58.3% (95% CI, 50.7–65.5%) in 1983–1994 to 40.0% (95% CI, 38.2–41.8%) in the period 2015–2019 [13]. In Japan, the prevalence was near 90% among individuals born before 1950, but with a subsequent decreasing trend, reaching less than 2% among the children born between 2000 and 2003 [14]. In a meta-analysis from Taiwan, of the 29 eligible studies including 38,597 subjects, the pooled prevalence of *H. pylori* infection decreased from 63.8% (95% CI, 55.9–71%) in 1990–2000 to 28.2% (95% CI, 21.8–35.6%) in 2016–2020 [15].

## 3. Risk Factors Associated with *Helicobacter pylori* Infection in Children

It is now well known that *H. pylori* infection is acquired mostly during childhood, mainly during the first decade of life [16]. Intrafamilial transmission is the main route, with potential transmission pathways including oral–oral, fecal–oral, and gastro–oral routes [8]. The global distribution of *H. pylori* exhibits significant variations, largely influenced by socio-economic status and overcrowding. Regions with lower and middle incomes exhibit a high prevalence ranging from 60% to 80%, attributed to factors such as crowded living conditions, inadequate sanitation facilities, and suboptimal hygiene practices. In sharp contrast, higher income countries present a considerably lower prevalence of *H. pylori*, ranging from 1.2% to 12.2%.

Results derived from a comprehensive review of seven cohort studies reveal the impact of birth country on the prevalence of *H. pylori* infection among asymptomatic children under the age of five. Infection rates within this group are documented at 20% to 40% in high-income countries and 30% to 50% in upper-middle-income countries.

Age and ethnicity are additional factors impacting *H. pylori* prevalence. Torres et al.’s study revealed higher infection rates among older children, ranging from 20% to 50% in those aged 0–5 years and 38% to 79% in older children [17].

Lower maternal education, lower education of both parents, larger family size, having parents, siblings infected with *H. pylori*, consuming meals in unsanitary conditions, and diarrhea are significantly associated with a higher prevalence of *H. pylori* infection detected by mixed tests in children [8].

## 4. Pathogenesis, Natural History, and Clinical Outcome of the Infection

### 4.1. Natural History

*H. pylori* is usually acquired in childhood, but infection persists lifelong without treatment. However, spontaneous eradication is described in infants or young children [18]. After successfully colonizing the gastric epithelium cells, chronic gastritis will develop in virtually all persistently colonized persons, but most of them will remain asymptomatic. The clinical course will depend on bacterial and host factors. Patients with a higher acid output are more likely to have gastritis predominantly in the antrum, which predisposes to duodenal ulcers [19]. Patients with a lower acid output are more likely to have gastritis in the body of the stomach, which predisposes them to gastric ulcers, and can initiate a sequence of events that, in rare cases, might lead to gastric carcinoma (0.5–2%) [16,20]. In addition, *H. pylori* infection induces the formation of mucosa-associated lymphoid tissue (MALT) in the gastric mucosa. Malignant lymphoma arising from MALT is another rare complication of *H. pylori* infection [21,22,23]. Who will develop the disease depends upon three factors: (a) the virulence of the infecting *H. pylori* strain, (b) the type and extent of the host immune response to infection, and (c) modulating cofactors such as geography, climate, gastrointestinal (GI) microbiota, medications, and diet [24].

### 4.2. Pathogenesis and Differences in Immune Response in Children and Adults

Key aspects of bacterial colonization involve flagellar motility, urease activity, mechanisms of adhesion, and damage to the gastric epithelium via vacuolization (vacuolating cytotoxin A-VacA). The *Helicobacter pylori* pathogenicity island exerts a key role in inflammation, composes a type IV secretory system (T4SS), and promotes the intracellular injection of cytotoxin-associated gene A (CagA) antigen. The host immune response is characterized by initial mucosal invasion with polymorphonuclear cells followed by activation of the innate and adaptive immune system with complex T helper 1 (TH1), TH17, and regulatory T (Treg) cell interactions [16].

Compared to adults, children with *H. pylori* colonization of the stomach have lower rates of gastroduodenal ulceration that have been attributed to a reduced polymorphonuclear and mononuclear cell infiltration [25]. Several studies have shown that infected children have reduced gastric inflammation compared to adults [25,26,27]. In addition, there is evidence that the immune response to infection in children is lower than in adults with lower mucosal recruitment of neutrophil, CD3+, and CD8+ cells as well as a lower activation status of NF-jB transcription [28]. Sequence analyses suggested that the *cagA* and *vacA* genes profiles of the bacteria isolated from infected adults and children might be comparable. Therefore, the lower level of inflammation in infected children contrasted with adults is not due to differences in bacterial strains or common virulence factors [29]. Children with *H. pylori* infection have a high Treg response but a low Th1 and Th17 response compared to adults [27,30]. Detecting *H. pylori*-specific Th17/Th1 in chronically infected adults may indicate that the initial regulatory response is gradually lost [31]. Consequently, Th1, Th17, and Treg results may imply gastric mucosal response to *H. pylori* [32]. Michalkiewicz et al. concluded that *H. pylori* infection in children was characterized by (a) Th1 expression profile, (b) lack of mRNA over-expression of natural immunity receptors, and (c) solid anti-inflammatory activities in the gastric mucosa, possibly due to increased activity of anti-inflammatory M2 macrophages [33]. A recent study involving 40 *H. pylori*-infected children and adults analyzed Th17A, Treg response, and gastric inflammation. FOXP3 cells expressed Tregs on antral mucosa and Th1 by Th17A expression. Both pathways were overexpressed in the mucosa of *H. pylori*-positive patients. Children presented an important regulatory response that inhibits inflammation (with higher FOXP3 cells) and significantly lower Th17A levels during *H. pylori* infection than adults [34].

### 4.3. Clinical Outcome

It is estimated that of the *H. pylori*-infected individuals developing chronic gastritis, approximately 90% will likely remain asymptomatic [35]. However, peptic ulcers and gastric cancer are associated with colonizing *H. pylori* in the stomach. These complications represent a weak percentage of *H. pylori*-infected individuals (15–20% for gastritis that might lead to ulcers and less than 2% for gastric cancer). Still, the burden is considerable as those two gastric diseases represent over a million global deaths annually [36,37]. *H. pylori* has been classified by the International Agency for Research on Cancer (IARC) as a group 1 (‘definite’) carcinogen since 1994 [38]. In 2015, at the Kyoto Consensus aiming in an aetiologic classification of chronic gastritis and duodenitis in adults and its appropriate diagnostic assessment, *H. pylori* gastritis was designated an infectious disease, meaning it should be treated whether or not associated with symptoms [39]. As of 2019, *H. pylori* gastritis as an infectious disease is included as a nosological entity in the 11th International Classification of Disease (ICD 11) [40].

The risk of ulceration increases when taking non-steroidal anti-inflammatory drugs (NSAIDs). In a meta-analysis, Huang et al. concluded that, compared to subjects not infected with *H. pylori* and not taking NSAIDs, the group positive for these two covariates had a relative risk of peptic ulcer disease 6.1 times higher [41]. The risk of duodenal ulcers is also increased by smoking [42]. These environmental factors are more critical in old age than in children or young adults.

*H. pylori* is additionally correlated with milder GI diseases such as non-ulcer dyspepsia and gastritis. Moreover, several non-GI diseases have been purportedly connected to *H. pylori* infection, such as iron deficiency anemia, diabetes mellitus, coronary artery diseases, and idiopathic thrombocytopenic purpura. However, it is important to note that the causal role of the bacterium in these associations is not well established. On the other hand, *H. pylori* infection is negatively associated with several upper GI diseases such as gastroesophageal reflux disease, Barret’s esophagus, eosinophilic esophagitis, and esophageal cancer, as well as non-GI diseases such as asthma, although evidence is still weak for these negative associations [43,44,45,46,47].

## 5. Clinical Manifestations in Children

The clinical manifestations of *H. pylori* infection in children are non-specific, and in some cases, they may be justified by the presence of complications. Although less frequent than in adults, *H. pylori*-associated gastroduodenal ulcers are responsible for abdominal pain and upper gastrointestinal bleeding [28]. Regarding *H. pylori* infection and gastritis, a meta-analysis of 75 studies with 5990 *H. pylori*-infected and 17,782 uninfected children evaluated histologic changes in the gastric mucosa according to the Updated Sydney System. *H. pylori*-positive children presented more cases with nodular gastritis and duodenal ulcer than *H. pylori*-negative children. The *H. pylori* infection was also associated with a higher relative risk for gastric antral and corpus chronic inflammation, the presence of neutrophils and lymphoid follicles, but rarely gastric mucosa atrophy. Intestinal metaplasia was only significantly higher in the antral area of patients with chronic active inflammation [48]. Kako et al. from Japan, in a study with mixed pediatric and adult patients, showed different histopathological patterns of gastritis across age categories. In pediatric patients, 84% had a pattern of nodular gastritis, present in 68% of the young adult group and only 8% of the older group. In the latter, inflammatory changes seem more pronounced in the gastric body than in the antrum with strong mucosal inflammation. Persistent nodular gastritis and mononuclear infiltrate may evolve into cancer or atrophy after years of a concurrent balance between Th1 and Th2 immune response [49].

On the other hand, trials in children investigating the role of *H. pylori* in non-ulcer dyspepsia are inconclusive because they are uncontrolled and of poor quality or do not include sufficient patients [50,51,52,53,54,55]. No clinical manifestations, especially non-recurrent abdominal pain (RAP), are specific to *H. pylori* infection in children [56]. In a meta-analysis published in 2010, a statistically significant association was documented only for epigastric pain and *H. pylori*, while vomiting, diarrhea, flatulence, chronic functional abdominal pain, halitosis, regurgitation, constipation, or nausea were not related to *H. pylori* infection. A more recent prospective observational study of 240 Brazilian children with chronic non-ulcer dyspepsia showed no association between GI symptoms and *H. pylori* infection except nausea [57]. Among 1558 children aged 6–13 years in Iran, 145 children with RAP, according to the Apley and Naish criteria, were compared with 145 age-matched healthy controls recruited from the same area. The symptoms of *H. pylori*-positive children were not significantly different from those of *H. pylori*-negative children [58].

A meta-analysis suggests that *H. pylori* may have immunoregulatory properties in Inflammatory Bowel Disease (IBD), and the inverse association seems stronger in pediatric patients and those with Crohn’s disease. Further studies are necessary to explore this subject [59,60]. Another meta-analysis supports the inverse relationship of infection with the risk of asthma, especially in CagA+ patients [61].

*H. pylori* infection is a causal factor in developing Iron Deficiency Anemia (IDA) [62]. Different pathways have been implicated, including decreased absorption of dietary iron due to hypochlorhydria, gastrointestinal blood loss, and enhanced uptake and sequestration of iron by the bacteria [63]. In children and adolescents with *H. pylori*-associated IDA, increased iron requirements play a significant role as a pathogen. On the other hand, bacterial virulence and host genetic factors remain to be investigated [62]. In some intervention studies, *H. pylori* eradication has been shown to reduce iron deficiency anemia [64,65,66,67].

Reports in children and adults suggest that when investigating causes of chronic immune thrombocytopenic purpura (ITP), *H. pylori* infection should be considered. Indeed, partial, or complete remission of thrombocytopenia has been reported in some patients after *H. pylori* eradication. *H. pylori* eradication has a significant therapeutic effect in patients with ITP, according to a meta-analysis of four pediatric and two adult randomized trials [68].

There is insufficient data to support the relationship between *H. pylori* infection and Henoch Schonlein purpura, coeliac disease, obstructive sleep apnea syndrome, and type I diabetes mellitus in children. On the other hand, a recent meta-analysis of 29 studies backs up the hypothesis that *H. pylori* infection is linked to growth defects in children, particularly height for age scores [69]. Emerging evidence suggests more and more extraintestinal pathologies to be related to *H. pylori* infection. However, most of them do not occur during childhood. Moreover, it seems that the development of extraintestinal manifestations because of *H. pylori* infection might also depend on other factors such as age, race, gender, and geographical areas. Therefore, elucidating the role of these demographic and host-related factors in the pathogenesis of *H. pylori*-associated extraintestinal manifestations should also be the focus of future research [70].

## 6. Diagnosis

Available diagnostic tools for *H. pylori* infection include both invasive and non-invasive methods. Using a gastric biopsy specimen obtained from upper gastrointestinal endoscopy, invasive tests include rapid urea testing (RUT), histology, culture, and molecular biology. The non-invasive methods include the ^13^C urease breath test (UBT), stool antigen test (SAT), serology, and molecular tests on non-invasive specimens (stool samples, saliva, and gastric juice).

Both invasive and non-invasive methods could be used in pediatrics. However, the primary objective of clinical investigation remains rooted in identifying the underlying cause of a child’s symptoms. Presently, existing evidence strongly suggests that an infection of *H. pylori* does not exhibit a direct link to symptoms in cases where peptic ulcer disease is absent. Consequently, pursuing a non-invasive test to identify the infection and subsequently initiate treatment upon a positive result is not deemed appropriate. As a result, the strategy known as ‘test and treat’, which hinges on employing non-invasive diagnostic methods to diagnose *H. pylori* infection in children, stands unrecommended and has never been endorsed.

In instances where children display symptoms that raise the suspicion of pathological conditions affecting the upper digestive tract, such as dyspepsia, a thorough evaluation involving upper-gastrointestinal endoscopy is recommended.

Thus, the joint European Society for Paediatric Gastroenterology, Hepatology, and Nutrition (ESPGHAN)/North American Society of Pediatric Gastroenterology, Hepatology and Nutrition (NASPGHAN) guidelines recommend the use of *H. pylori*-positive culture or a combination of histology and one another biopsy-based test such as RUT or polymerase chain reaction (PCR) [4].

The isolation of *H. pylori* via culture is deemed the gold standard technique, given its attainment of 100% specificity [4]. Nonetheless, it is characterized by its demanding nature, influenced by factors such as meticulous pre-analytical conditions and the necessity for a well-equipped microbiology laboratory, which ultimately curtails its widespread applicability. As an alternative, molecular-based methods might be favored for identifying the infection and generating antimicrobial susceptibility profiles. Moreover, all the invasive methods require discontinuing proton pump inhibitors (PPIs) for at least 2 weeks and antibiotics for at least 4 weeks preceding endoscopy [4,71].

The diagnostic accuracy of histology varies with bacterial load and the examiner’s experience. Fortunately, the incorporation of immunohistochemistry, which is progressively becoming more accessible in laboratories, substantially bolsters the sensitivity and specificity of histological assessments [72]. Alongside its capacity to detect the bacteria, histology furnishes invaluable insights into the condition of the gastric mucosa, a conclusion supported by recent findings from a meta-analysis conducted in a pediatric population [48].

Molecular methods are less often used to diagnose *H. pylori* infection in pediatrics. However, a Turkish study found 94% concordance between PCR and histology for *H. pylori* diagnosis in children [73]. In a more recent study, Bogiel et al. demonstrated the robust accuracy of the PCR-based approach compared to histology in a group of 104 children [74]. RUT is strongly recommended in Japanese guidelines to diagnose active *H. pylori* infection in children [75]. However, the utility of RUT remains constrained in various regions owing to the unavailability of commercial assays [76].

In addition to the classical methods, endoscopic findings could be helpful in the diagnosis strategy. An analysis of 13 studies comparing endoscopic methods to standard diagnostic approaches (culture, RUT, UBT, SAT, and serology) for *H. pylori* infection found that the presence of Regular Arrangement of Collecting venules (RACs) was a highly significant predictor of *H. pylori* absence (OR: 55, sensitivity 78.3%, and specificity 93.8%) [77], and a subsequent systematic review with 4070 patients confirmed this exclusion criterion with even higher specificity (97%) [78], suggesting its potential utility in combination with traditional diagnostic methods, particularly when RAC results are negative. This could be helpful in combination with classical diagnostic tools, especially in cases where RAC is negative.

As in adults, it is recommended to monitor the success of eradication in children at least 4 weeks after the end of the treatment. For this purpose, non-invasive tests are indicated, particularly UBT or SAT. PPIs should be discontinued for at least 2 weeks (and antimicrobial agents for 4 weeks) as they interfere with the sensitivity of UBT and SAT [4,75]. A historical pan-European consensus including 18 countries has resulted in a practical guideline for an optimal and harmonized use of UBT from the prescription to the interpretation of the results [79]. SAT is less sensitive than UBT; nevertheless, it is the easiest method for children regarding sample collection and the most accessible for countries with reduced incomes. Many recent studies have confirmed the reliability of various SATs for *H. pylori* diagnosis [80,81].

Serology is not recommended for diagnosing *H. pylori* infection in children, neither for initial diagnosis nor eradication control due to a lower sensitivity. Its use is, therefore, limited to epidemiological studies [4,75].

## 7. Antimicrobial Susceptibility Testing

Culture-based methods and molecular tests provide both diagnosis and susceptibility testing. Unlike culture-based methods, molecular methods are often limited to one (clarithromycin) or two antibiotics (clarithromycin and levofloxacin). Nevertheless, molecular techniques are increasingly used due to the culture’s time-consuming and fastidious nature [82]. Feng et al. found that the success of tailored therapy based on clarithromycin susceptibility is significantly higher when employing the molecular detection of point mutations (92%) in comparison to the phenotypic method (70.4%) [83].

The most promising advances in recent years for the child population involve the combination of diagnosis and susceptibility testing in stool samples, saliva, and gastric juice using various molecular methods [1,70,80,84].

## 8. Antibiotic Resistance

The widespread use of antibiotics provides *H. pylori* with the opportunity to develop various resistance mechanisms, with point mutations being the predominant occurrences [82,85]. Table 1 below summarizes some recent data on primary resistance to antibiotics obtained worldwide in pediatrics during the last years [73,86,87,88,89,90,91,92]. Amoxicillin resistance is rare (0–1%) in most of the studies, except in Germany (20%) and Vietnam (50%), but these last data are questionable and need additional confirmation. Except for the Belgian series, clarithromycin primary resistance is dramatically high (more than 20%), with a maximum of resistance observed respectively in China (45%), Poland (54%), and Vietnam (92%). Quinolones are not generally recommended for children. However, we observe a very high rate (31%) of resistance in some studies, notably in Germany and Vietnam. Primary metronidazole resistance rates are highest in Asian countries, probably related to the most frequent use of this drug to cure parasitic infections. Tetracycline resistance is rare (null in most of the studies), except in the German (12%) and Poland (4.5%) series.

Borka Balas et al. [93] and Boyanova et al. [94] recently reviewed the evolution of *H. pylori* resistance to antibiotics in different countries and regions worldwide, including pediatric population and mixed adults and children, respectively. The general trend in children is stability for amoxicillin and tetracycline and increasing resistance rates for clarithromycin, metronidazole, and fluoroquinolones. Nevertheless, an increase in resistance to tetracycline in Iran (3% [1999–2000], 12% [2011–2016] and 18% [2017–2019]), to amoxicillin in Iran (9% [1999–2000], 14% [2011–2016 and 36% [2017–2019]), and in Bulgaria (4.2% [2007–2014] and 8.2% [2015–2021]) can be noted. We are also surprised by the decreased resistance rates to metronidazole found in Chile.

**Secondary resistance:** Previous attempts at eradication is a well-known risk factor to the development of antibiotic resistance. This phenomenon is underscored by data from the EuroPedHp registry, revealing substantially higher resistance rates—51.4% for clarithromycin, 40% for metronidazole, and 25.5% for the combination of clarithromycin and metronidazole—when compared to the relatively lower rates of 25%, 17.7%, and 3.8% observed for primary resistance to the same antibiotics [87].

These observations are consistent with similar trends observed in other independent studies [78,93,94], reinforcing the significance of prior eradication attempts in driving increased antibiotic resistance rates. Addressing this challenge is crucial for informing antimicrobial stewardship strategies and public health interventions aimed at curbing the emergence and dissemination of antibiotic-resistant pathogens.

**Multidrug resistance:** In line with the dramatic increase in *H. pylori* resistance to the major antibiotics used for eradication, multidrug resistance in children is also a topic of increasing concern. A systematic review and meta-analysis published in 2022 further underscored this concern, revealing an aggregated prevalence of primary multidrug-resistant *H. pylori* strains isolated in children at 6.0% (95% CI, 3.1–11.6%) [95]. This finding emphasizes the urgency of robust surveillance and strategic intervention to manage and mitigate the spread of multidrug resistance, thereby protecting the efficacy of treatment regimens for pediatric *H. pylori* infections.

**Heteroresistance:** Heteroresistance is characterized by the simultaneous presence of both resistant and susceptible pathogens within the same patient, and it can possibly lead to underestimating antimicrobial resistance. In their comprehensive meta-analysis of 22 studies, Lopo et al. reported a documented prevalence of 6.8% (95% CI, 5.1–8.6%) for clarithromycin heteroresistance and 13.8% (95% CI, 8.9–18.6%) for metronidazole heteroresistance [96]. Furthermore, research conducted on children by Gosciniak et al. indicated notable discrepancies of 20% for clarithromycin and 13.3% for metronidazole. The findings from the Belgian study conducted in 2021 revealed heteroresistance rates of 1.8% for clarithromycin, 3.5% for levofloxacin, and 10.5% for metronidazole among pediatric patients [86]. These results collectively emphasize the importance of adopting more sensitive diagnostic techniques to accurately detect and quantify heteroresistance, ensuring effective antimicrobial stewardship and better-informed clinical decision-making to address the challenges posed by diverse resistance profiles within individual patients.

**Comparison to adults:** In general, when compared to the adult population, *H. pylori* resistance rates in infected children tend to be lower. A meta-analysis including 178 studies across 65 countries worldwide confirmed this trend with only a few notable exceptions. These exceptions include higher metronidazole resistance rates in children compared to adults in the Eastern Mediterranean region (81% versus 61%) and in America (40% versus 22%), elevated clarithromycin resistance in children compared to adults in the Western Pacific region (85% versus 32%), and surprisingly, increased levofloxacin resistance among children in the Eastern Mediterranean region compared to adults (29% versus 18%) [97].

## 9. Treatment

In contrast to the adult guidelines, which recommended the eradication of *H. pylori* in all patients with an infection (regardless of the presence of peptic ulcer disease or background risk of gastric cancer), the pediatric guidelines take a different approach. The accepted indications for eradication treatment in children are gastro–duodenal ulcer disease, infection if a first-degree relative has gastric cancer, and refractory IDA. However, the definition of refractory IDA is imprecise and subject to various interpretations. The ‘test and treat’ strategy have not been recommended in pediatrics. However, if an infection by *H. pylori* is found during an upper GI endoscopy performed for suspicion of an organic disease but no ulcer (or erosion) is visualized, treatment can be offered after adequate discussion with the child and their family. In such cases, it is essential to address the possible side effects linked to treatment and the absence of proven benefits [4].

The goal of treatment is to achieve at least 90% eradication on a per-protocol basis on the first attempt [98]. A high eradication rate prevents the development of antibiotic resistance, the spread of resistant strains of *H. pylori* in the population, and reduces the number of re-treatments and eradication controls. The standard of care since the late 1990s, in children, is the combination of two antibiotics (mostly amoxicillin combined with either clarithromycin or metronidazole) and a proton pump inhibitor [99,100]. This treatment should be given for 14 days, as clearly emphasized in the adult consensus guidelines [71] and the latest pediatric consensus guidelines [4]. Since the frequency of resistance is increasing worldwide as shown in the section ‘Antibiotic resistance’ of this manuscript, determination of antimicrobial susceptibility is requested to tailor the eradication scheme. Indeed, recent studies showed that empirical triple therapy, especially when containing clarithromycin, performs poorly [91,101,102,103,104,105]. The meta-analysis of Wen et al. also showed an eradication rate of 71% with empirical triple therapy containing either clarithromycin or metronidazole [106]. On the contrary, using a tailored triple therapy containing clarithromycin or metronidazole for 14 days, the eradication rate obtained in the most recent multicenter registry reach the target of 90% (95% CI, 87–93%) [87]. These studies suggest that empirical treatment should no longer be used.

In the absence of available antimicrobial susceptibility testing, or in case of resistance to both clarithromycin and metronidazole, the recommendation in pediatrics suggests using an empirical triple therapy for 14 days combining a proton pump inhibitor with amoxicillin and metronidazole. Studies published during the last 5 years with this treatment have shown, however, substantial variability in the eradication rate but all remain substantially below the target of 90% [101,102,103,107,108]. With a duration of 14 days and a treatment tailored to antimicrobial susceptibility, the eradication rate obtained in the most recently published registry reached 92% [87].

Other regimens have been proposed such as sequential treatment consisting of two successive treatment periods of 5 days: amoxicillin and a proton pump inhibitor in two doses per day then a combination of metronidazole and clarithromycin with a proton pump inhibitor [109]. The rationale for using this sequential treatment is that amoxicillin would reduce the bacterial load (thus the risk of mutations) and destroy the bacterial cell wall during the first 5 days. Subsequently, the intracellular diffusion of clarithromycin may be increased. However, this treatment has not been proven to be more effective than a triple therapy for 14 days [87,110] and it exposes the child to three different antimicrobial agents, plus a proton pump inhibitor, which may induce more adverse events and more antimicrobial resistance in the case of treatment failure. The same is true for concomitant quadruple therapy without bismuth, which has not been adequately studied in children.

Bismuth-based therapies were frequently used in the 80s and the 90s before being abandoned for more than a decade. However, such combinations have been re-introduced after 2011 [71] and are even recommended as a first-line therapy in adults when available. Bismuth-based quadruple therapies containing tetracycline have not been properly studied in children and, due to the possible side effects, are not indicated before adulthood. Recent data in children exist with quadruple therapies containing bismuth sub-citrate, a proton pump inhibitor, amoxicillin, and metronidazole, and seem to be very efficient. The more recent prospective open cross-sectional study of 288 Chinese children comparing four different regiments showed that this treatment given for 14 days allowed the reaching of 90%, superior to 74% with standard triple therapy [111]. In a prospective single-arm trial in Belgium in 36 children, the same treatment given for only 10 days also showed a very high eradication rate (97%) [112]. Another recently published study proposes a sequential 7-day proton pump inhibitor with amoxicillin followed by a 7-day proton pump inhibitor, tetracycline, metronidazole, and bismuth subsalicylate in Turkish adolescents (mean age 15.1 ± 2.4 years) with a high eradication rate (92%) [113]. Finally, in Vietnam, in a prospective trial involving 237 children treated with different tailored regimens, 43 children infected with multidrug-resistant *H. pylori* strains received 14 days of bismuth-based quadruple therapy and the eradication rate of the bismuth quadruple scheme was 88% [114].

## 10. Rescue Treatment

Providing there is an adequate indication for treatment, a rescue treatment should be proposed in case of eradication failure. Compliance to the previous treatment must be assessed since this is a major cause of failure [108]. Previously used antibiotics, especially clarithromycin and metronidazole, should be avoided from the rescue regimen given the high likelihood of resistance. Nevertheless, the success of rescue treatment seems to be lower, as shown by a multicenter registry performed in Europe where success of a rescue treatment was only 59% [87]. Tetracycline or fluoroquinolones should be avoided in children and certainly used with extreme precautions. Tetracycline can cause yellow discoloration, hypoplasia of the enamel, and bone deposits. Fluroquinolones are associated with disabling side effects involving tendons, muscles, joints, nerves, and the central nervous system. These side effects can occur hours to weeks after exposure and may potentially be permanent.

## 11. Conclusions

In recent years, a growing interest has emerged in understanding *H. pylori* infection among children. Despite indications of a declining trend, the prevalence of *H. pylori* infection remains markedly high among both children and adolescents globally, with variations based on location and sanitation standards. Addressing this challenge requires concerted efforts to enhance hygiene, sanitary conditions, and access to clean water sources, thereby reducing the burden of *H pylori* infection on a global level.

New insights have been uncovered regarding lower mucosal immune responses and the possible role of *H. pylori* in preventing immune disorders such as allergies. Further research is needed to explore immune responses and clinical manifestations in children and to develop targeted interventions to reduce the burden of *H. pylori*-related diseases in pediatric populations.

Clinical investigation should prioritize identifying the cause of abdominal symptoms instead of solely focusing on detecting *H. pylori* infection. Unfortunately, in practical situations, it is not always possible to adhere to the guidelines. Failing to eradicate the infection during the first-line treatment not only reduces the chance of success with rescue therapy but also leads to secondary antimicrobial resistance and increase healthcare costs. To avoid these challenges, consensus guidelines advocate for an upper gastrointestinal endoscopic examination with at least six gastric biopsy samples for histology and culture, or PCR.

Over the last years, many studies have highlighted a concerning surge in antimicrobial resistance rates in children across various countries, especially the frequency of multidrug resistance. Considering this, we recommend standard antibiotic susceptibility testing of *H. pylori* in pediatric patients prior to initiating antibiotic therapy. All pediatric guidelines emphasize genotypic methods (PCR-based) over relying solely on phenotypic (culture-based) approaches for tailored eradication therapy due to their higher analytic performance and resilience to gastric biopsy conditions. However, their cost remains a limiting factor, and their availability for a broader spectrum of antibiotics remains limited.

In conclusion, considering all the aspects discussed above, we anticipate that this review will contribute to a reduction in the use of inappropriate tests (non-invasive tests such as *H. pylori* serology or breath tests) as diagnostic methods and while eradication treatment is necessary for children with peptic ulcer disease, it may not be warranted for all detected cases. As antibiotic resistance rates rise and treatment options in the pediatric population remain limited, persistent research is imperative to understand disease pathogenesis and develop effective prevention and treatment strategies for children affected by the infection. Additionally, when devising national preventive strategies, factors such as socioeconomic status, living conditions, local population eradication rates, and antimicrobial susceptibility profiles should all be considered.

## Figures and Tables

**Table 1:** Recent worldwide data on primary antibiotic resistance of *Helicobacter pylori* in pediatric populations over the past few years.

First Author, Year	Region	Period	N	AMO (%)	CLA (%)	LEV (%)	MET (%)	TET (%)	RIF (%)	CLA-MET (%)	CLA-LEV (%)	CLA-MET-LEV (%)	Comments
Miendje Deyi et al., 2023	Belgium	January–December 2021	72	0	13.9	13.9	43.1	0	-	8.3	1.4	1.4	*1
Le Thi et al., 2022	Europe	2017–2020	651	1	25	5.4	17.7		8.5	3.8			
Geng et al., 2022	China (Chongqing)	March–July 2020	112	0	47.3	18.8	88.4	0	-	30.4	1.8	10.7	
Shu et al., 2022	China (Southeast)	March 2015–December 2020	1638	0	32.8	22.8	81.7	0	-	16.4	1.8	9%	*2
Helmbold et al., 2022	Germany	October 2015–July 2019	51	20	45	31	59	12	22	22.4	-	-	
Su et al., 2022	Taiwan	January 2009–August 2019	70	2	23.1	8.2	20		-	-	-	-	
Li et al., 2021	China (Southwest)	2019	53	0	45.3	15.1	73.6	0	60.4	28.3 (including CLA-MET RIF)	-	9.4 (+Rif)	*3
Van Thieu et al., 2021	Vietnam	November 2019–June 2020	76	50	92.1	31.6	14.5	0	-	13.2			*4
Krzyzek et al., 2020	Poland	2016–2018	22	0	54.5	9.1	31.8	4.5	-	27.3	4.5	4.5	*5
Guven et al., 2019	Turkey	December 2016–April 2018	93	-	27	15	-	-	-				*6

AMO: amoxicillin; CLA: clarithromycin; LEV: levofloxacin; MET: metronidazole; TET: tetracycline; RIF: rifampicin; *1: lower overall resistance compared to adults; *2: MET-LEV:10.8%; *3: MET-LEV:10.8%; *4: AST method not detailed, and the authors fail to explain the remarkably high AMO resistance rate; *5: higher CLA resistance compared to adults; *6: concordance histology and PCR 94%.

## Data Availability

Not applicable.
